# Antimicrobial Activity of *Lacticaseibacillus rhamnosus* CRL 2244 Extracts Against Community- and Hospital-Acquired *Staphylococcus aureus*

**DOI:** 10.3390/antibiotics14080812

**Published:** 2025-08-08

**Authors:** Cecilia Rodriguez, Briea Gasca, Vyanka Mezcord, Robert A. Bonomo, Gauri Rao, Nicholas T. Salzameda, Maria Soledad Ramirez

**Affiliations:** 1 Centro de Referencia para Lactobacilos (CERELA), Consejo Nacional de Investigaciones Científicas y Técnicas (CONICET), Tucuman 4000, Argentina; crodriguez@cerela.org.ar; 2 Center for Applied Biotechnology Studies, Department of Biological Science, College of Natural Sciences and Mathematics, California State University Fullerton, Fullerton, CA 92831, USA; bsgasca@csu.fullerton.edu (B.G.); mezcordvyanka@csu.fullerton.edu (V.M.); 3 Research Service and GRECC, Louis Stokes Cleveland Department of Veterans Affairs Medical Center, Cleveland, OH 44105, USA; robert.bonomo@va.gov; 4 Departments of Medicine, Pharmacology, Molecular Biology and Microbiology, Biochemistry, Proteomics and Bioinformatics, School of Medicine, Case Western Reserve University, Cleveland, OH 44105, USA; 5 Cleveland Veteran Affair Medical Center for Antimicrobial Resistance and Epidemiology (Case VA CARES), Case Western Reserve University, Cleveland, OH 44105, USA; 6 Department of Clinical Pharmacy, Alfred E. Mann School of Pharmacy and Pharmaceutical Sciences, University of Southern California, Los Angeles, CA 90033, USA; gaurirao@usc.edu; 7 Department of Chemistry and Biochemistry, College of Natural Science and Mathematics, California State University Fullerton, Fullerton, CA 92831, USA; nsalzameda@fullerton.edu

**Keywords:** *Staphylococcus aureus*, lactic acid bacteria, probiotics, methicillin-resistant, transcriptional response, secreted compounds

## Abstract

**Background/Objectives:** Methicillin-resistant *Staphylococcus aureus* (MRSA) remains a critical public health concern due to its multidrug resistance and capacity to form persistent infections, particularly in the context of implanted medical devices. Alternative therapeutic strategies that target bacterial virulence instead of viability are increasingly explored. This study aimed to evaluate the antimicrobial and antivirulence activity of an extract derived from *Lacticaseibacillus rhamnosus* CRL 2244 against two MRSA strains—USA300 and M86—and to elucidate its effects on bacterial physiology and gene expression under host-mimicking conditions. **Methods**: Antimicrobial activity was assessed using agar diffusion, MIC, and time-kill assays. Scanning electron microscopy of cells exposed to the extract confirmed decreased cellular density and morphological changes. Phenotypic assays evaluated biofilm formation, staphyloxanthin production, and adhesion to fibronectin. RT-qPCR analyzed transcriptional responses. Viability was assessed in the presence of human serum and type I collagen. **Results**: The CRL 2244 extract demonstrated bactericidal activity with up to 6-log_10_ CFU/mL reduction at 1× MIC. In USA300, the extract reduced the expression of *hla*, *lukAB*, *fnbA*, and *icaA*, correlating with decreased staphyloxanthin levels. In M86, a significant reduction in biofilm formation and repression of *lukAB*, *nucA*, and *fnbA* were observed. Adhesion to fibronectin was impaired in both strains. The extract showed no cytotoxicity in human serum but reduced viability in collagen-enriched conditions. **Conclusions**: The *Lcb. rhamnosus* CRL 2244 extract modulates MRSA virulence in a strain-specific manner, targeting key regulatory and structural genes without inducing cytotoxic effects.

## 1. Introduction

Bacterial resistance to antibiotic represents one of the most pressing global health challenges of the 21st century [1]. The increase in infections caused by resistant pathogens not only limits therapeutic options, but also increases morbidity, mortality, and healthcare costs. Among these pathogens, methicillin-resistant *Staphylococcus aureus* (MRSA) has been designated as a critical priority by both the World Health Organization (WHO) and the Centers for Disease Control and Prevention (CDC) due to its high prevalence and clinical impact [1,2,3].

MRSA is responsible for a wide range of acute and chronic infections, both hospital-acquired (HA-MRSA) and community-acquired (CA-MRSA). While HA-MRSA is a major cause of pneumonia, bloodstream infections, and surgical site infections, CA-MRSA is mainly associated with skin and soft tissue infections, but can also cause serious conditions such as osteomyelitis and toxic shock syndrome [4,5,6]. In addition, MRSA plays a key role in chronic lung infections in patients with cystic fibrosis, where its ability to form biofilms and acquire antibiotic-tolerant phenotypes makes it difficult to eradicate and worsens patient outcomes [7,8]. The pathogenic success of *S. aureus* lies in its diverse arsenal of virulence factors that facilitate colonization, immune evasion, and tissue invasion. In particular, Panton–Valentine leukocidin (PVL), which is more frequently associated with CA-MRSA strains, induces neutrophil lysis and contributes to tissue necrosis. Enterotoxins and toxic shock syndrome toxin-1 (TSST-1) are implicated in foodborne illness and systemic toxicity. In addition, adhesin expression and biofilm-forming ability enable persistence in host tissues and medical devices, further enhancing resistance. Protein A, coagulase, and capsular polysaccharides also contribute to immune evasion and virulence [9,10,11,12].

The global propagation of MRSA strains resistant to multiple antibiotic classes has prompted an urgent search for alternative antimicrobial strategies [13,14]. Among these, lactic acid bacteria (LAB) have shown promise due to their Generally Recognized as Safe (GRAS) status, natural antimicrobial activity, and their health-promoting effects, particularly in maintaining gut health and modulating the immune system [15,16]. LAB are well-known producers of diverse bioactive metabolites, including organic acids, peptides, and bacteriocin-like molecules with antimicrobial activity, and recent studies have reported their ability to inhibit multidrug-resistant pathogens, including MRSA [17,18,19]. Our group recently demonstrated that *Lacticaseibacillus rhamnosus* CRL 2244 secretes antimicrobial compound(s) with bactericidal activity against carbapenem-resistant *Acinetobacter baumannii* (CRAB) as well as other clinically relevant pathogens, including MRSA [20]. Notably, these compounds also act synergistically with β-lactam antibiotics and vancomycin, restoring the susceptibility of *S. aureus* strains resistant to these drugs [20].

Based on these findings, the present study investigates the antimicrobial activity of the extract from *Lcb. rhamnosus* CRL 2244 against MRSA strains, with emphasis on its potential clinical application. We employed both phenotypic and transcriptomic approaches to assess the bactericidal effects of the extract on two representative MRSA strains: the epidemic community-associated USA300 clone (characterized by SCC*mec* type IVc and the PVL gene) [21,22] and the hospital-associated strain M86, isolated from a cystic fibrosis patient [23]. In addition, we evaluated its efficacy in physiologically relevant conditions, such as human serum, collagen matrices, and fibronectin-binding assays targeting a host protein crucial for *S. aureus* colonization. Although *Lactobacillus* species are not natural colonizers of the respiratory tract, there is interest in their secreted metabolites due to their potential use as purified compounds or fractions, independent of the live bacteria, as novel therapeutic options. This study therefore provides new insights into the antimicrobial properties of these metabolites as a first step toward their possible clinical application after appropriate purification and analysis, highlighting the therapeutic potential of LAB-derived antimicrobials against multidrug-resistant pathogens in clinically complex infection scenarios.

## 2. Results

### 2.1. Antimicrobial Activity of Lcb. rhamnosus CRL 2244 Extract Against MRSA Strains

The antimicrobial activity of the extract obtained from *Lcb. rhamnosus* CRL 2244 against MRSA strains was evaluated by spot-on-the-lawn assay (Table 1). In CLDE medium, the extract exhibited low to moderate inhibitory activity, with inhibition halos of 10 mm for strain CA-MRSA USA300 and 12 mm for HA-MRSA M86. In contrast, high activity was observed on blood agar, with halos of 20 mm for both strains, suggesting that blood components enhance the bioactive compound’s effectiveness. This effect could be attributed to the blood agar components that promote diffusion or stability of the active compound. Likewise, the activity of the extract was evaluated against isogenic mutants of USA300 in virulence-related genes using CLDE plates. Inhibition halos ranging from 15 to 20 mm were observed, with the greatest effect recorded for the Δ*hla* mutant (IDH = 20 mm), suggesting that the response to the extract could be influenced by pathogen-specific virulence factors (Table 1).

The minimum inhibitory concentration (MIC) of the *Lcb. rhamnosus* CRL 2244 extract was determined against MRSA strains USA300 and M86. The MIC value for both strains was found to be 5 µg/µL, indicating that this concentration was sufficient to inhibit visible bacterial growth after 24 h of incubation.

### 2.2. Morphological Changes Induced by Lcb. rhamnosus CRL 2244 Extract on S. aureus Cells

**Figure 1:**
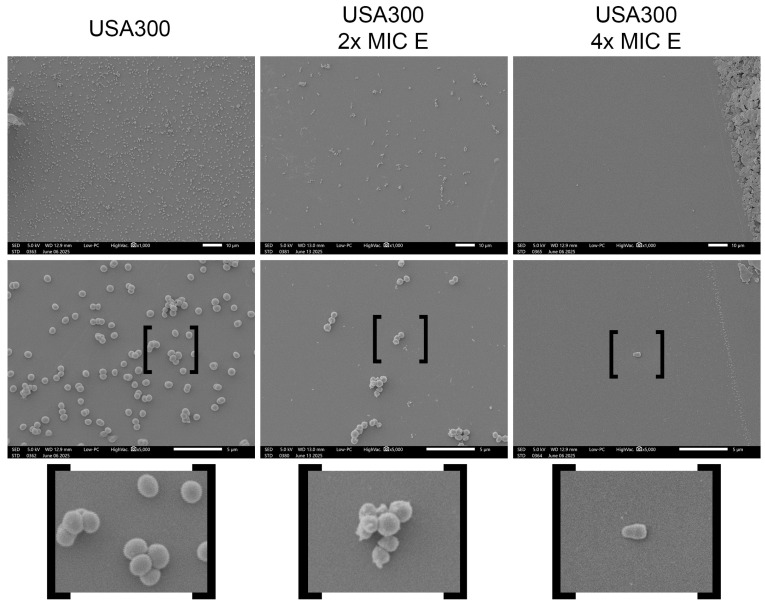
Scanning electron microscopy (SEM) analysis of *S. aureus* USA300 exposed to the CRL 2244 extract. Representative SEM micrographs of *S. aureus* USA300 cells grown under control conditions (labeled as USA300), and after treatment with 2× MIC (USA300 2× MIC E) or 4× MIC (USA300 4× MIC E) of the extract. All images were taken at 1000×, and 5000× magnification; scale bars represent 10 μm and 5 μm.

In addition, microscopy analysis and colony counting were performed to assess morphological alterations and reductions in cellular density in *S. aureus* cultures exposed to the extract. Treatment of *S. aureus* USA300 with the extract at 2× and 4× MIC resulted in noticeable morphological changes and a marked decrease in bacterial cell density, confirming the extract’s ability to impair bacterial growth (Figure 1; Figure S1). Under control conditions, bacterial cells appeared in dense aggregates with smooth surfaces and intact coccoid morphology (Figure 1). In contrast, treatment with the extract at 2× MIC led to a reduction in cell density, with fewer cells (Figure 1; Figure S1). Cells exposed to 2× MIC showed morphological irregularities, suggesting membrane stress or early structural compromise (Figure 1). At 4× MIC, these effects were markedly intensified. Only few cells sparsely distributed were seen, with visible signs of morphological changes, in particular an increase in cell length after exposure to the extract (Figure 1). These findings indicate that the extract alters cell structure in a dose-dependent manner, potentially compromising bacterial viability, further confirmed by quantitative CFU assays (Figure 1; Figure S1).

### 2.3. Bactericidal Effect of Lcb. rhamnosus CRL 2244 Extract Against MRSA Strains

**Figure 2:**
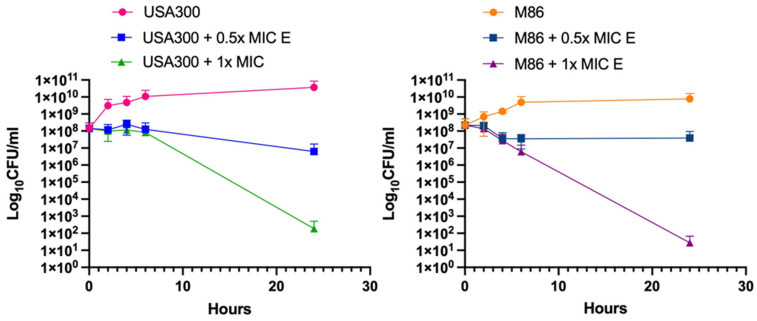
*Lcb. rhamnosus* CRL 2244 extract killing activity against MRSA. The strains USA300 and M86 were incubated at 37 °C for 24 h in presence of extract at 0.5× MIC and 1× MIC. CFU/mL were determined at different incubation times for a period of 24 h. All assays were carried out in technical duplicates.

To evaluate the impact of *Lcb. rhamnosus* CRL 2244 extract (E) on the viability of the two MRSA strains, USA300 and M86, time-kill assays were performed (Figure 2). In both cases, bacterial cultures treated with 1× MIC of the extract showed a marked decrease in viable cell count over time. In the case of strain USA300, treatment with 1× MIC resulted in a reduction of ~6 log_10_ CFU/mL after 24 h compared to the untreated control, indicating a potent bactericidal effect (Figure 2). A similar trend was observed for strain M86, with bacterial counts below the limit of detection at 24 h post-treatment. This confirms the extract’s potent bactericidal activity against both MRSA strains. In contrast, treatments with 0.5× MIC of the extract led to a moderate decrease in bacterial load, maintaining bacteriostatic activity for the same period (Figure 2). Untreated controls for both strains showed exponential growth, confirming the antimicrobial efficacy of the CRL 2244 extract in a both dose- and time-dependent manner.

To evaluate the potential synergistic or additive effect of the CRL 2244 extract in combination with a β-lactam antibiotic, a killing assay was performed using subinhibitory concentrations of ampicillin. A standardized inoculum of *S. aureus* USA300 was cultured in the presence of 0.5× MIC ampicillin alone or in combination with 0.5× MIC of the extract. Bacterial viability was assessed at 0, 2, 4, 6, and 24 h. As shown in Figure S2A, treatment with ampicillin resulted in a ~4 log_10_ reduction in CFU/mL over time, whereas the combination treatment led to a more pronounced decrease in bacterial viability (Figure S2A), supporting a potential additive interaction between the extract and ampicillin. Interestingly, in addition to reduced viability, cultures treated with the combination of ampicillin and extract also displayed a small colony phenotype (Figure S2B).

### 2.4. MRSA Transcriptional Changes Triggered by the Extract from Lcb. rhamnosus CRL 2244

**Figure 3:**
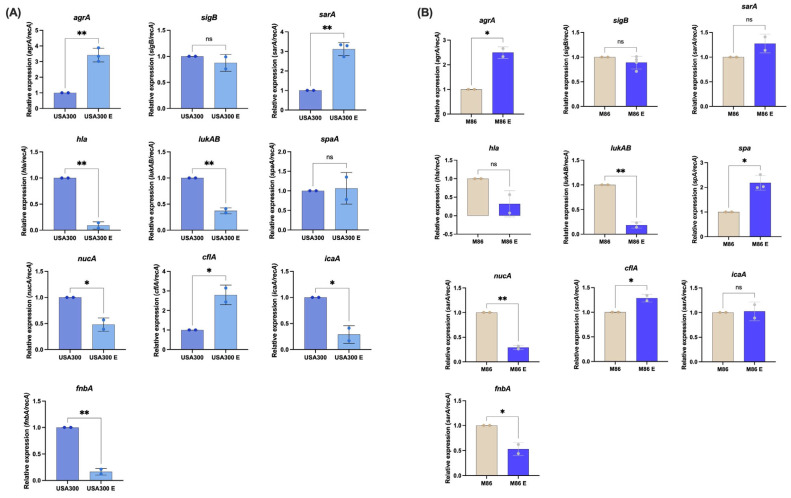
Differential gene expression profiles of MRSA strains to *Lcb. rhamnosus* CRL 2244 extract. The clinical isolates USA300 (**A**) and M86 (**B**) were grown with and without sub-MIC concentration of extract from *Lcb. rhamnosus* CRL 2244 for 18 h at 37 °C. The differential gene expression was determined by qRT-PCR of genes involved in quorum sensing (*agrA*), global transcriptional regulation (*sarA* and *sigB*), immune evasion (*spaA*), cytotoxin production (*hla* and *lukAB*), biofilm formation (*icaA*), surface adhesion (*fnbA* and *clfA*), and nucleic acid degradation (*nucA*), all of which are key factors in *S. aureus* virulence. The data presented are the mean ± standard deviation (SD) of normalized relative to *recA* transcript levels calculated using the qBASE method. Statistical significance (*p* < 0.05) was determined by one-way ANOVA followed by *t*-test. * *p* < 0.05; ** *p* < 0.005; ns, not significant. Experimental data were obtained from technical triplicates of three independent biological replicates. Error bars indicate SD.

To assess the transcriptional response of MRSA to the CRL 2244 extract, we performed quantitative RT-PCR (qRT-PCR) on the CA-MRSA strain USA300 and the HA-MRSA strain M86 following exposure to sub-inhibitory concentrations (sub-MIC) of the extract (Figure 3). The results revealed significant transcriptional changes in several virulence-associated genes, particularly in USA300. In USA300, the exposure to the extract (USA300 E) resulted in strong upregulation of *agrA*, *sarA*, and *cflA* (*p* < 0.01 for *agrA* and *sarA*) compared to the untreated control (Figure 3A). *agrA* is part of the *agr* quorum sensing system that regulates virulence, while *sarA* plays a major role in regulation of biofilm formation. *clfA*, which encodes clumping factor A, also showed increased expression, possibly reflecting a compensatory response aimed at enhancing adhesion in the face of biofilm disruption. In contrast, genes associated with cytotoxin production, including *hla* (α-hemolysin) and *lukAB* (leucocidin), were significantly downregulated, suggesting that the extract may reduce the cytotoxic potential of *S. aureus*. *nucA*, which encodes a thermonuclease involved in biofilm matrix degradation, was also downregulated, possibly reflecting an alteration in biofilm structure. Furthermore, key adhesion-related genes, *fnbA* and *icaA*, were significantly downregulated in response to the extract (Figure 3A), indicating that the extract interferes with major biofilm formation pathways, likely weakening *S. aureus* colonization and persistence. Interestingly, no significant differences were detected in the expression levels of *sigB*, a general stress response regulator, and *spaA,* associated with immune evasion, suggesting that the extract primarily affects virulence and biofilm regulation rather than general stress response pathways.

In the HA-MRSA M86 strain (Figure 3B), qRT-PCR analysis revealed a distinct transcriptional response to the extract compared to USA300. Significant upregulation was observed in *agrA*, *spa*, and *cflA* (Figure 3B), while *lukAB*, *nucA*, and *fnbA* were significantly downregulated (Figure 3B). No changes were detected in the transcript’s levels of *sigB*, *sarA*, *hla*, and *icaA*, indicating a more moderate and strain-specific response compared to USA300.

Taken together, these findings demonstrate that exposure to the CRL 2244 extract modulates key virulence-related genes in both MRSA strains, with USA300 exhibiting a broader and more pronounced transcriptional shift. This suggests that the extract may differentially impact MRSA strains depending on their genetic background and virulence profile, highlighting its potential as a strain-specific anti-virulence approach.

### 2.5. Effect of the Extract on MRSA Virulence: Inhibition of Biofilm Formation and Staphyloxanthin Production

**Figure 4:**
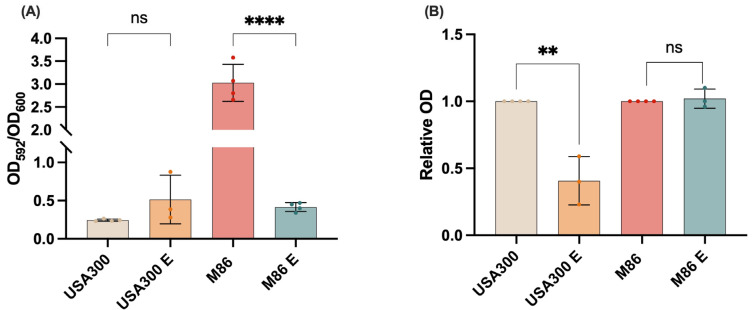
Effect of *Lcb. rhamnosus* CRL 2244 extract on virulence features of MRSA strains USA300 and M86. (**A**) Biofilm formation (**B**) Relative staphyloxanthin production. Data represent mean ± SD from three independent experiments. Statistical significance was determined by one-way ANOVA with post hoc test (ns = not significant; ** *p* < 0.01; **** *p* < 0.0001).

The biofilm-forming ability of MRSA strains USA300 and M86 was evaluated under untreated and extract-treated conditions (USA300 E and M86 E, respectively). Biofilm quantification, expressed as the OD_592_/OD_600_ ratio, showed that strain M86 produced significantly more biofilm compared to the other conditions analyzed (Figure 4A). However, exposure to the CRL 2244 extract (M86 E) drastically reduced its biofilm formation, bringing levels down to those observed in USA300 and USA300 E (Figure 4A). No significant differences were detected between USA300 and its extract-treated condition (Figure 4A), indicating that the extract has a more pronounced antibiofilm effect on high biofilm-forming strains like M86. These findings suggest that the extract specifically targets and disrupts robust biofilm architectures, likely interfering with structural components or regulatory pathways involved in biofilm development. This selective effect on high biofilm-forming strains may be advantageous for controlling persistent infections.

These assays were conducted in LB medium, a nutrient-rich environment commonly used for bacterial growth, and the observed effects were also confirmed in the more enriched Terrific Broth (TB), supporting the robustness and reproducibility of the extract’s antibiofilm activity. 

Staphyloxanthin production was also evaluated in the same experimental conditions. Compared to the untreated strain, USA300 exposed to the extract (USA300 E) exhibited a significant reduction in pigment production (Figure 4B). In contrast, no significant difference in staphyloxanthin production was observed between M86 and its treated counterpart (M86 E) (Figure 4B). Changes in pigment production may reflect strain-specific metabolic shifts in *S. aureus*, which may vary depending on the strain’s baseline levels and susceptibility to the treatment. Taken together with the biofilm data, these findings underscore a strain-dependent response to the CRL 2244 extract, with effects observed on multiple virulence-associated phenotypes.

### 2.6. Impact of the Lcb. rhamnosus CRL 2244 Extract on MRSA Viability in Host-Mimicking Conditions

**Figure 5:**
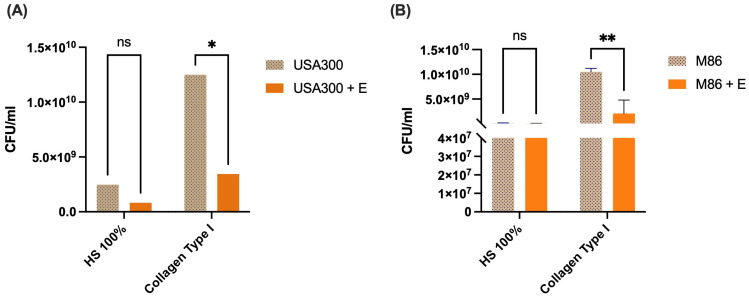
Effect of *Lcb. rhamnosus* CRL 2244 extract on MRSA survival in human serum and collagen-supplemented medium. Viability of MRSA strains USA300 (**A**) and M86 (**B**) was evaluated after exposure to extract (E) under two physiologically relevant conditions: 100% human serum (HS) and LB broth supplemented with type I collagen (8 µg/mL). Bacterial survival was expressed as log_10_ CFU/mL. Bars represent mean ± standard deviation of three independent experiments. Statistical significance was determined using a two-tailed unpaired *t*-test. * *p* < 0.01; ** *p* < 0.01; ns, not significant.

The antimicrobial activity of the CRL 2244 extract was evaluated under physiologically relevant conditions using MRSA strains USA300 and M86 (Figure 5). In 100% human serum, no significant differences in bacterial counts were observed between untreated and extract-treated cultures for either strain. This lack of activity could be linked to the extract’s reduced solubility soluble in serum, which could interfere with its antimicrobial efficacy. In contrast, exposure to the extract in type I collagen-supplemented medium resulted in a significant reduction in bacterial survival. For strain USA300, extract exposure led in a moderate but statistically significant decrease in CFU/mL (Figure 5A). A more pronounced effect was seen in M86, with approximately a 1-log decrease in CFU/mL (Figure 5B). These findings suggest that the bactericidal effect of the extract is condition-dependent, with enhanced effectiveness in collagen-rich environments. This may be due to interactions between type I collagen and bacterial surface adhesins, potentially facilitating increased susceptibility to the bioactive compounds in the extract. These results support the potential of CRL 2244-derived antimicrobials to function in host-like settings where extracellular matrix components play a key role in bacterial colonization.

### 2.7. Effect of the Lcb. rhamnosus CRL 2244 Extract on Bacterial Adhesion of MRSA Strains

**Figure 6:**
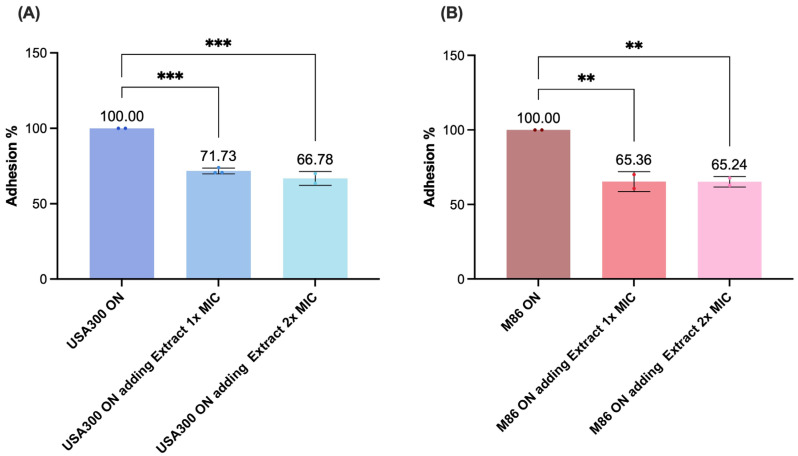
Effect of the extract on adhesion of MRSA (**A**) USA300 and (**B**) M86 strains. Fibronectin-coated wells were inoculated with bacterial cultures incubated under different experimental conditions: co-treatment during the 2 h adhesion assay, in which overnight cultures together with extract at 1× MIC or 2× MIC and untreated control (overnight cultures in LB) were added to the wells. Adhesion was quantified by OD_405_ measurements after 2 h incubation at 37 °C. The results are expressed as absolute adhesion values (% relative to control). Data represent mean ± SD of three independent experiments. Statistical significance was determined using one-way ANOVA with Tukey’s post hoc test: ** *p* < 0.01, *** *p* < 0.001.

To assess the impact of the *Lcb. rhamnosus CRL* 2244 extract on the adhesion of MRSA strains USA300 and M86, fibronectin-coated wells were inoculated with bacterial cultures in the presence of the extract at two different MIC concentrations (co-treatment) and in its absence (control). Simultaneous exposure to the extract at 1× MIC and 2× MIC during the adhesion phase significantly reduced bacterial attachment for both strains (Figure 6). For USA300, adhesion was reduced by 28.27% and 26.86% at 1× MIC and 2× MIC, respectively (Figure 6A). M86 showed similar reductions of 27.62% and 28.98% under the same treatment conditions (Figure 6B). Both strains exhibited maximal adhesion in the untreated control group (100%), which served as a baseline for comparisons in the other experimental conditions (Figure 6A,B). Therefore, the extract significantly impairs MRSA adhesion to fibronectin, a key step in colonization.

## 3. Discussion

In a context of the growing threat posed by infections caused by multidrug-resistant pathogens such as MRSA, compounds derived from LAB have been postulated as innovative therapeutic alternatives. Their safety, metabolic diversity, and ability to modulate virulence mechanisms without exerting direct selective pressure on bacterial viability make them attractive candidates for antivirulence strategies [24,25,26,27]. In particular, metabolites produced by LAB have shown inhibitory effects against multidrug-resistant clinical strains by impairing adhesion, interfering with biofilm-forming, and modulating the expression of virulence factor-associated genes, which is especially relevant in clinical settings involving implantable devices, such as heart valves or catheters, which promote colonization and persistent infections [28,29,30].

In this study, we demonstrated that the extract derived from *Lcb. rhamnosus* CRL 2244 exerts a modulatory effect on MRSA USA300 and M86 strains at both the transcriptional and phenotypic levels, notably reducing staphyloxanthin production and adhesion capacity under host-mimicking conditions. Although the chemical identity of the active compound(s) remains undefined, the findings presented here represent a key step toward functional characterization and support the potential of CRL 2244 extract as an antivirulence agent for preventing or treating MRSA infections in clinical settings. According to the ISAPP consensus [31,32], purified microbial metabolites in the absence of microbial biomass do not meet the definition of postbiotics or paraprobiotics. In our study, the antimicrobial activity is due to a polar metabolite secreted by *Lcb. rhamnosus* CRL 2244 without detectable cells or cellular fragments [20]; therefore, once identified, it should be referred to by its specific chemical name rather than considered a postbiotic. Importantly, this metabolite maintains activity despite mild variations in pH or temperature and upon exposure to proteolytic or hydrolytic enzymes [20], supporting its physiological stability and robustness, key attributes that reinforce its potential as a therapeutic candidate.

The extract showed potent antimicrobial activity against both community-associated (USA300) and hospital-associated (M86) MRSA strains, consistent with prior observations in CRAB strains [20,33], supporting the hypothesis of a broad-spectrum mechanism of action. The enhanced inhibition observed on blood agar plates suggests that its components may influence the diffusion and stability of bioactive molecules, as well as promote MRSA growth and susceptibility, as previously reported [34,35,36]. Interestingly, the increased susceptibility of the isogenic USA300 Δ*hla* mutant points to a link between virulence factors and extract sensitivity. *hla*, which encodes α-hemolysin, plays a central role in *S. aureus* cytotoxicity and immune evasion [37]. Its deletion may reduce the pathogen’s capacity to counteract the stress imposed by the extract, as similarly observed with other antimicrobials targeting virulence factors [38]. Time-kill assays validated that the extract’s bactericidal activity, with reductions of up to 6 log_10_ CFU/mL after 24 h at 1× MIC in both strains, similar to previous results with CRAB strains [33]. Consistent with this potent bactericidal effect, SEM analysis revealed morphological alterations in *S. aureus* cells exposed to the extract, including a reduction in cell density and an increase in cell length, features previously observed in other multidrug-resistant pathogens treated with this extract [29]. These structural changes suggest a mechanism of action involving alteration of the cell envelope and were found to be dose-dependent, in agreement with the activity of the extract observed in CFU and MIC assays.

At the molecular level, the extract induced transcriptional changes consistent with virulence modulation. In USA300, we observed a broader transcriptional response than M86. USA300 showed overexpression of *agrA* and *sarA*, two master regulators of quorum sensing and biofilm formation, respectively. Despite this, expression of critical virulence genes—including *hla*, *lukAB*, *icaA*, and *fnbA*—was significantly reduced. This apparent decoupling between regulatory elements and effector genes suggests a reprogramming of the virulence profile rather than a global activation, a phenomenon previously reported in response to environmental or metabolic stimuli [38]. This is consistent with reports showing metabolic stress impairing *agr* network function and altering virulence gene expression [39]. Such dysregulation could involve feedback inhibition or interference with quorum-sensing signaling, ultimately leading to upregulation of *agrA* and *sarA* but downregulation of their target genes.

Phenotypically, USA300 did not exhibit significant biofilm changes, likely due to its low basal biofilm formation under the experimental conditions in LB and TB media at 37 °C for 48 h without shaking, which were identical for both strains. In contrast, staphyloxanthin production was markedly reduced, correlating with *hla* repression and potentially a less oxidative cellular environment. As a key antioxidant carotenoid, staphyloxanthin protects *S. aureus* from host-derived stress [40,41]. Reduction in staphyloxanthin, possibly mediated by *sarA* repression, could increase the vulnerability of the pathogen, reinforcing its value as an antivirulence target [42,43]. In line with this hypothesis, prior studies have shown that natural compounds and anti-inflammatory drugs can disrupt staphyloxanthin biosynthesis [44].

The M86, by contrast, showed a more restricted transcriptional response. While *sarA*, *hla*, and *icaA* expression remained unchanged, we observed increased expression of *agrA* and *spa* and repression of *lukAB*, *nucA*, and *fnbA*. These molecular changes correlated with a pronounced reduction in biofilm formation, suggesting the extract may be particularly effective against high biofilm-forming strains—a trait also observed in CRAB [29]. This difference may be partially explained by the distinct genetic backgrounds and clinical origins of USA300 (ST8, SCC*mec* IV, PVL-positive) and M86 (ST5, linezolid-resistant, PVL-positive isolate from a cystic fibrosis patient) that likely contribute to this differential antibiofilm response [21,23]. Consistent with our findings, several studies have reported antibiofilm effects of *Lcb. rhamnosus* against *S. aureus*. Biosurfactants produced by *Lcb. rhamnosus* and *L. jensenii* inhibited adhesion and disrupted MRSA biofilm structure at concentrations of 25–50 mg/mL [45]. Similarly, in vitro assays using urinary devices showed that *Lcb. rhamnosus* could displace preformed biofilms of *S. aureus* and *Escherichia coli* through metabolite release and possible integration into the biofilm matrix [46]. Complementary findings include extracts from mature *Lcb. rhamnosus* GG biofilms removed up to 67% of *S. aureus* biofilms at six-fold concentration with bactericidal effects up to 99.9% [47] and ultrasonicated extracts from *Lcb. rhamnosus* YT showing a dose-dependent and stable antibiofilm effect mediated by polysaccharides and proteins [48].

The downregulation of *nucA*, involved in extracellular DNA degradation and biofilm dispersal [49], may reflect alterations in biofilm matrix maturation or stability. In contrast to USA300, M86 showed no changes in levels of staphyloxanthin production, likely due to stable expression of biosynthesis-related genes such as *sigB* or *crtM*, which were not analyzed in this study.

When tested under host-mimicking conditions, the CRL 2244 extract’s activity varied. In human serum, no significant reduction in bacterial viability was detected, suggesting low cytotoxicity in protein-rich environments. However, a significant decrease in cell viability was observed in the presence of type I collagen, particularly for M86. This indicates that certain components of the tissue microenvironment may sensitize MRSA to the effect of the extract, in agreement with studies showing that interaction with extracellular matrix proteins modulates virulence and antimicrobial susceptibility of *S. aureus* [9,50]. Adhesion assays using fibronectin-coated surfaces showed that the extract impaired MRSA anchoring, even at subinhibitory concentration, showing a strong effect in the highly adherent M86 strain. This phenotype correlates with reduced expression of key adhesion genes such as *fnbA* and *icaA*, supporting the hypothesis that CRL 2244 extract interferes with early colonization—an essential step in staphylococcal pathogenesis and biofilm-associated infections [38].

Finally, like transcriptomic studies by Peng et al. (2023) who showed that *Lcb. rhamnosus* SCB0119 modulates key genes in *E. coli* and *S. aureus*, we observed repression of *hla*, *lukAB*, and *fnbA*, along with functional reductions in adhesion and staphyloxanthin production [51]. These findings support the concept that LAB-derived compounds can reprogram virulence traits in a strain-dependent manner, reinforcing their value as antivirulence rather than bactericidal agents.

## 4. Materials and Methods

### 4.1. Bacterial Strains and Growth Conditions

USA300 (CA-MRSA) [21,52] and M86 (HA-MRSA) [23] strains were used in this work. In addition, a total of six *S. aureus* (SA) USA 300 mutants were used for spot-on-the-lawn assays to test antimicrobial activity (Table 1). The mutants were in *agrA, sarA, lukS-PV*, *cflA*, *fnbA* (Nebraska Tn Mutant Library, distributed by BEI Resources), and *hla* [53]. The strains were grown on Cystine-Lactose Electrolyte-Deficient (CLED) medium (Beckton Dickinson, Franklin Lakes, NJ, USA) overnight at 37 °C and used within 24 h.

### 4.2. Preparation of Extracts from Lcb. rhamnosus CRL 2244

To obtain the crude cell-free extract, *Lcb. rhamnosus* CRL 2244 was first grown in MRS broth (2% *v*/*v* inoculum) and incubated at 37 °C for 96 h without agitation. After incubation, the cell free conditioning medium (CFCM) was obtained and used for extract preparation. The culture was filtered and immediately subjected to lyophilization using a Virtis 4K benchtop lyophilizer. The lyophilized material was resuspended in 250 mL of deionized water and extracted by liquid–liquid partitioning with ethyl acetate (1:1 *v*/*v*) in a 2000 mL separatory funnel. The mixture was shaken vigorously and allowed to separate; the organic phase was collected. The aqueous phase was re-extracted twice with decreasing volumes of ethyl acetate (200 mL and 190 mL). The combined organic extracts were dried with ~30 g anhydrous magnesium sulfate, filtered, and concentrated using a rotary evaporator (IKA RV 10 digital V) set at 150 rpm with gradually reduced pressure from 395 mbar to 75 mbar over 20–30 min. The concentrated extract was stored at −20 °C and used without further purification.

### 4.3. Antimicrobial Susceptibility Assays

The antimicrobial activity of the *Lcb. rhamnosus* CRL 2244 extract was assessed using a modified version of the well diffusion method outlined in a previous study [20]. Bacterial strains were resuspended in sterile saline solution (0.85% NaCl, *w*/*v*) to a concentration of 0.5 McFarland units (1.5 × 10^8^ CFU/mL). These suspensions were then inoculated onto CLED and blood agar plates; blood agar testing was performed only for a representative subset of mutants as a complementary evaluation. A volume of 10 μL of the extract (800 μg/μL) was applied to the surface of the plates. After incubating at 37 °C for 24 h, the inhibition zone diameters (IDHs) were measured. The results were categorized as follows: less active (IDH ≤ 10 mm), moderately active (IDH = 11–14 mm), and highly active (IDH ≥ 15 mm) [54]. All assays were conducted using three independent biological replicates.

The minimum inhibitory concentration (MIC) of the extract was determined against MRSA strains USA300 and M86 by broth microdilution following CLSI guidelines [55]. Briefly, serial two-fold dilutions of the extract were prepared in cation-adjusted Mueller–Hinton (CAMH) broth in 96-well microplates. Bacterial suspensions were adjusted to approximately 5 × 10^5^ CFU/mL and added to each well. Plates were incubated at 37 °C for 24 h, and bacterial growth was assessed visually. The MIC was defined as the lowest extract concentration that completely inhibited visible growth.

To ensure that the observed activity was attributable to the CRL 2244 extract and not to the solvent, 10% DMSO without extract was included as a vehicle control in all assays. For validation of the MIC and agar diffusion methods, vancomycin was used as a positive antibiotic control, and *S. aureus* ATCC 29213 served as a reference strain. Untreated bacterial cultures were included as growth controls.

### 4.4. Scanning Electron Microscopy (SEM)

To evaluate the reduction in cellular density and morphological changes induced by the extract, SEM was performed on cultures of strain USA300. A standardized inoculum (0.5 McFarland), prepared from colonies grown overnight on LB agar at 37 °C, was used to inoculate 2 mL of LB broth supplemented with the extract at 2× and 4× MIC. As a control, USA300 was cultured under identical conditions in LB broth without the addition of the extract. After incubation for 18–20 h at 37 °C, the cells were centrifuged at 5000 rpm for 5 min, washed twice with sterile saline solution, and fixed in 2% glutaraldehyde solution. Cells were deposited on protamine coated glace slides, dehydrated in an ethanol series, and critical-point-dried for subsequent visualization at the core imaging Lab, Department of Biological Science, CSUF with the JCM-7000 NeoScope™ Benchtop SEM (Portsmouth, NH, USA).

Bacterial growth of the samples, before fixation, was assessed by serial dilution and colony counting.

### 4.5. Time-Killing Assay

The bactericidal effect of the extract was evaluated against MRSA USA300 and M86 strains for a period of 24 h, following a previously described protocol [20]. The assays were performed in tubes using a bacterial inoculum prepared by 1:10 diluting a 0.5 McFarland (1.5 × 10^8^ CFU/mL) suspension in Luria–Bertani (LB) broth. Cultures were exposed to the extract at concentrations of 0.5× MIC and 1× MIC, while untreated cultures (no extract) were used as controls.

Samples were incubated at 37 °C with shaking, and bacterial viability was assessed at 0, 2, 4, 6, and 24 h by serial dilutions and cultures on CLDE agar. Colony forming units (CFUs) were counted after overnight incubation at 37 °C. Each condition was tested in two independent experiments, performed in duplicates. In addition, a time-killing assay with a 0.5× MIC of ampicillin and 0.5× MIC of ampicillin plus 0.5× MIC of the extract was performed to observe synergy or additive effects.

### 4.6. RNA Extraction and Quantitative Reverse Transcription Polymerase Chain Reaction (qRT-PCR) Assays

MRSA strains USA 300 and M86 cells were cultured in the absence or presence of 2.5 µg/µL of extract (sub-MIC concentration = 0.5× MIC) for 18 h at 37 °C. Total RNA was extracted in triplicate for each condition using a commercial kit (Direct-zol RNA Kit, ZymoResearch), following treatment with 10 mg/mL of Lysostaphin (Sigma-Aldrich, St. Louis, MO, USA) and 50 mM EDTA buffer for 1 h at 37 °C. DNase-treated RNA was then used for complementary DNA (cDNA) synthesis with the iScript™ Reverse Transcription Supermix (Bio-Rad, Hercules, CA, USA), following the manufacturer’s instructions. The cDNA concentrations were adjusted to 50 ng/µL, and qPCR was performed using qPCRBIO SyGreen Blue Mix Lo-ROX, according to the manufacturer’s guidelines (PCR Biosystems, Wayne, PA, USA). Each qPCR assay included at least three biological replicates of cDNA and was performed in triplicate using the CFX96 Touch™ Real-Time PCR Detection System (Bio-Rad, Hercules, CA, USA). The data were presented as NRQs (normalized relative quantities), calculated using the qBASE method [56,57], with *recA* genes as normalization controls. Experimental data were obtained from technical triplicates of three independent biological replicates. Statistically significant differences were indicated by asterisks and determined by ANOVA followed by Tukey’s multiple comparison test (*p* < 0.05) using GraphPad Prism Version 10.5.0 (GraphPad Software).

### 4.7. Biofilm Formation Assay

Biofilm formation in MRSA strains USA300 and M86 was assessed using Terrific Broth (TB) and Luria–Bertani (LB) medium to enhance stress conditions. The assay was adapted from the protocol described by Rodriguez et al., 2023 [33], with modifications to perform it in test tubes. Bacterial cultures were prepared under two conditions: a control group consisting of 1.5 mL of LB medium and 150 µL of bacterial suspension adjusted to 0.5 McFarland, and a treatment group with the same inoculum supplemented with the extract at 0.5× MIC. Samples were incubated at 37 °C for 48 h without shaking to promote biofilm development.

After incubation, planktonic cells were removed, and the remaining biofilm was stained with 1% crystal violet solution for 30 min. Excess dye was discarded, and tubes were washed twice with 1× PBS. Samples were then air-dried, and the retained stain was solubilized with 30% (*v*/*v*) acetic acid for 30 min. Optical density was measured at 592 nm. Total biomass was previously determined by OD600 before washing, and results were expressed as the ratio between biofilm biomass and total biomass (OD592/OD600).

All experiments were performed in triplicate. Statistical analysis was conducted using the Mann–Whitney U test in GraphPad Prism Version 10.5.0 (GraphPad Software, San Diego, CA, USA), with a significance threshold set at *p* < 0.05.

### 4.8. Staphyloxanthin Production Assay

The production of staphyloxanthin in MRSA strains USA300 and M86 was assessed following the protocol described by Morikawa (2021) [58]. Briefly, the strains were inoculated in terrific broth and Luria–Bertani (LB) broth, with and without exposure to a sub-MIC concentration (2.5 µg/µL) of the extract and incubated at 37 °C for 48 h with agitation. Subsequently, 850 µL of the culture was collected by centrifugation at 10,000× *g* for 1 min, washed with distilled water, and resuspended in 200 µL of methanol. The samples were then heated at 55 °C for 5 min and centrifuged at 15,000× *g* for 1 min to remove cellular debris. The extraction process was performed twice, and the resulting extracts were pooled into a single tube, adjusting the final volume to 1 mL with methanol. Finally, absorbance was measured at 465 nm.

The relative absorbance (RA) was calculated using the equation RA = A_treated/A_control. The results were interpreted as follows: RA = 1 indicates no effect of the antimicrobial on staphyloxanthin production; RA < 1 suggests a reduction in its production; and RA > 1 may indicate either an unexpected effect or an antimicrobial-induced increase in production.

### 4.9. Human Fluids Survival Assay

The effect of the extract on bacterial survival in human fluids was evaluated in MRSA strains USA300 and M86. Assays were conducted using two conditions: 100% human serum (Innovative Research, Novi, MI, USA, certified vendor approved by ISO, FDA, USDA, and EPA) and LB broth supplemented with type I collagen (8 µg/mL physiological concentration, Calbiochem Inc., San Diego, CA, USA). In both cases, cultures were prepared in a final volume of 1 mL, containing 100 µL of an overnight bacterial culture (adjusted to 0.5 McFarland), with or without the addition of extract at 1× MIC (5.85 µg/mL final concentration). Samples were incubated overnight at 37 °C with agitation. Bacterial viability was determined by serial dilution and plating on CLDE agar to calculate CFU/mL. Each condition was tested in two independent experiments, performed in duplicate.

### 4.10. Adhesion Assay

The effect of the extract on bacterial adhesion of MRSA strains USA300 and M86 was evaluated using 96-well microplates coated with Corning^®^ BioCoat^®^ Fibronectin Clear Flat Bottom TC (Thomas Scientific, Swedesboro, NJ, USA), following the protocol described by Peacock et al. (2000) [59]. Fibronectin-coated wells were inoculated with 100 µL of bacterial suspensions in LB broth incubated overnight under the following conditions: untreated control (overnight cultures in LB) and co-treatment during adhesion, in which overnight cultures were added together with extract at 1× MIC or 2× MIC directly into the wells. After inoculation, all plates were incubated for 2 h at 37 °C. After incubation, the wells were washed three times with 1× PBS, fixed with 2% glutaraldehyde for 1 h, stained with 1% crystal violet for 5 min, rinsed with distilled water, and air dried. The stain was then solubilized with 30% acetic acid and absorbance was measured at 405 nm to quantify adherent biomass. Adhesion was expressed as relative adhesion ratio (AR = A_treated/A_control) and as percentage adhesion relative to control (% Adhesion = [A_treated/A_control] × 100). All conditions were tested in triplicate in at least two independent experiments.

### 4.11. Statistical Analysis

All experiments were performed in technical duplicate or triplicate and repeated in at least three independent biological replicates, unless otherwise indicated. Data are expressed as mean ± standard deviation (SD). Statistical analyses were performed with GraphPad Prism Version 10.5.0, applying one-way or two-way ANOVA depending on the experimental design. Where appropriate, Tukey’s multiple comparison test or unpair two-tailed Student’s *t*-test was used as post hoc analysis. A *p*-value < 0.05 was considered statistically significant. Significance levels are indicated in the legend of each figure as follows: * *p* < 0.05; ** *p* < 0.01; *** *p* < 0.001; **** *p* < 0.0001; ns = not significant.

## 5. Conclusions

The results of this study underscore the therapeutic potential of compounds produced by LAB, particularly the extract derived from *Lcb. rhamnosus* CRL 2244. Rather than functioning as a conventional antimicrobial, this extract modulates *S. aureus* virulence by targeting key regulatory pathways in a strain-dependent manner. Given the increasing prevalence of multidrug-resistant *S. aureus* infections and the limited number of effective therapeutic options, these findings underscore the importance of developing alternative therapeutic approaches. Further in vivo studies are essential to assess the clinical efficacy, safety, and potential effect on bacterial adaptation associated with long-term use of this extract.

## Figures and Tables

**Table 1:** Antimicrobial activity of *Lacticaseibacillus rhamnosus* CRL 2244 extract against *S. aureus* strains tested by the spot-on-the-lawn method.

Strains	Special Features	IDH (mm)	
		CLDE Agar	Blood Agar
*Staphylococcus aureus*			
M86	Linezolid Resistance, PVL+	10	20
USA 300	*mec*A*,* PVL+	12	20
SAUSA 300 mutants			
1992	Δ*agrA* (accessory gene regulator protein A)	16	19
0605	Δ*sarA* (accessory regulator A)	15	nd
1382	Δ*lukS-PV* (Panton–Valentine leukocidin, LukS-PV	16	nd
0772	Δ*clfA* (clumping factor A)	18	nd
2441	Δ*fnbA* (fibronectin binding protein A)	16	nd
α-toxin	Δ*hla* (α-toxin)	20	20

IDH: inhibition diameter halos.

## Data Availability

Data are contained within the article and supplementary materials.

## Supplementary Materials

The following supporting information can be downloaded at: https://www.mdpi.com/article/10.3390/antibiotics14080812/s1, Figure S1. Viability of *S. aureus* USA300 cultured in LB broth and supplemented with 2× and 4× MIC of the extract determined by serial dilution and CFU count before fixation for SEM analysis; Figure S2. (A) Time-kill assay evaluating the effect of CRL 2244 extract in combination with ampicillin against *S. aureus* USA300. Viability was assessed by CFU enumeration at 0, 2, 4, 6, and 24 h. Data represent the mean ± SD of biological replicates. (B) Treatment of USA300 cultures with the combination of ampicillin and the extract resulted in the appearance of small colony variants.
