# Supplementary figures and images for: Antimicrobial Activity of Lacticaseibacillus rhamnosus CRL 2244 Extracts Against Community- and Hospital-Acquired Staphylococcus aureus
Source: Antibiotics (Basel). 2025 Aug 8;14(8):812. doi: 10.3390/antibiotics14080812 (PMC12382898; doi:10.3390/antibiotics14080812)

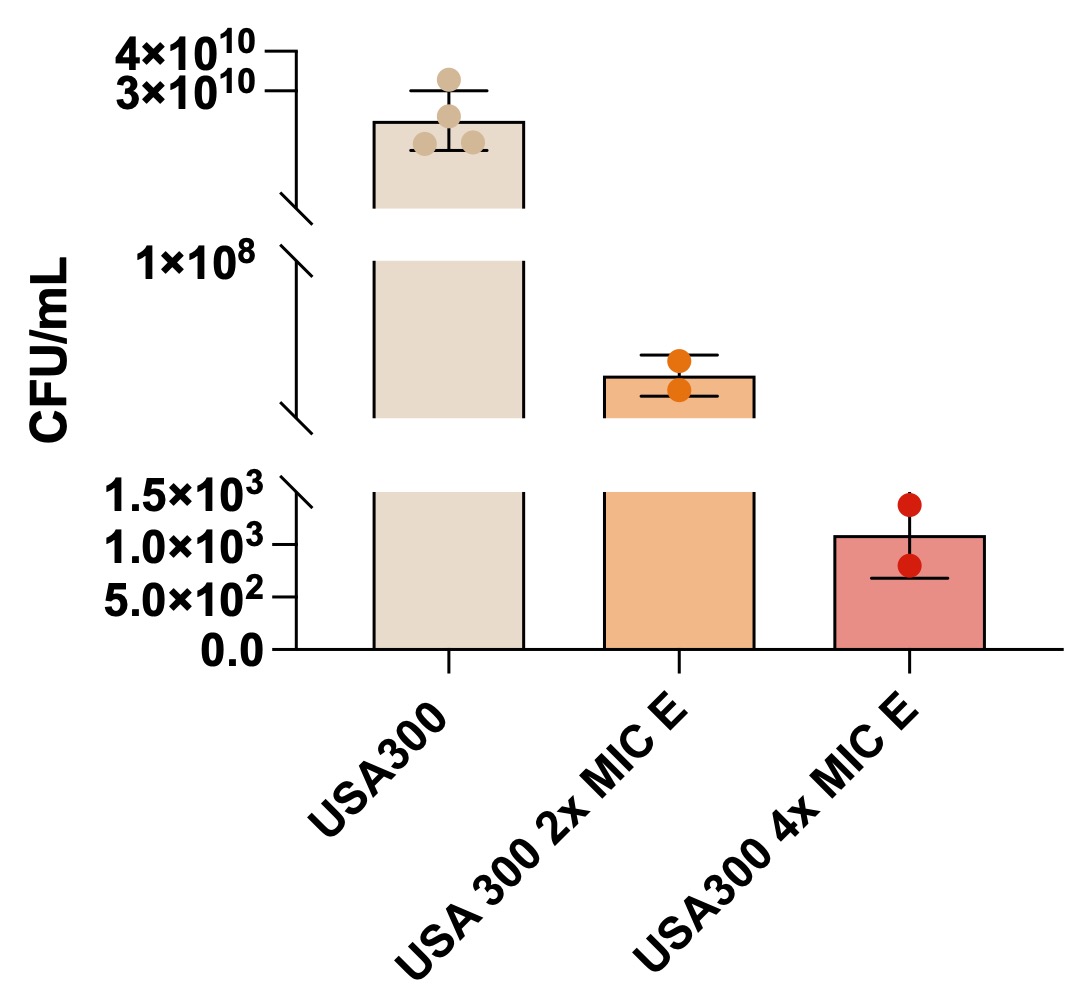

Supplement: Supplementary file 1 [file antibiotics-14-00812-s001.zip › Figure S1.jpg]

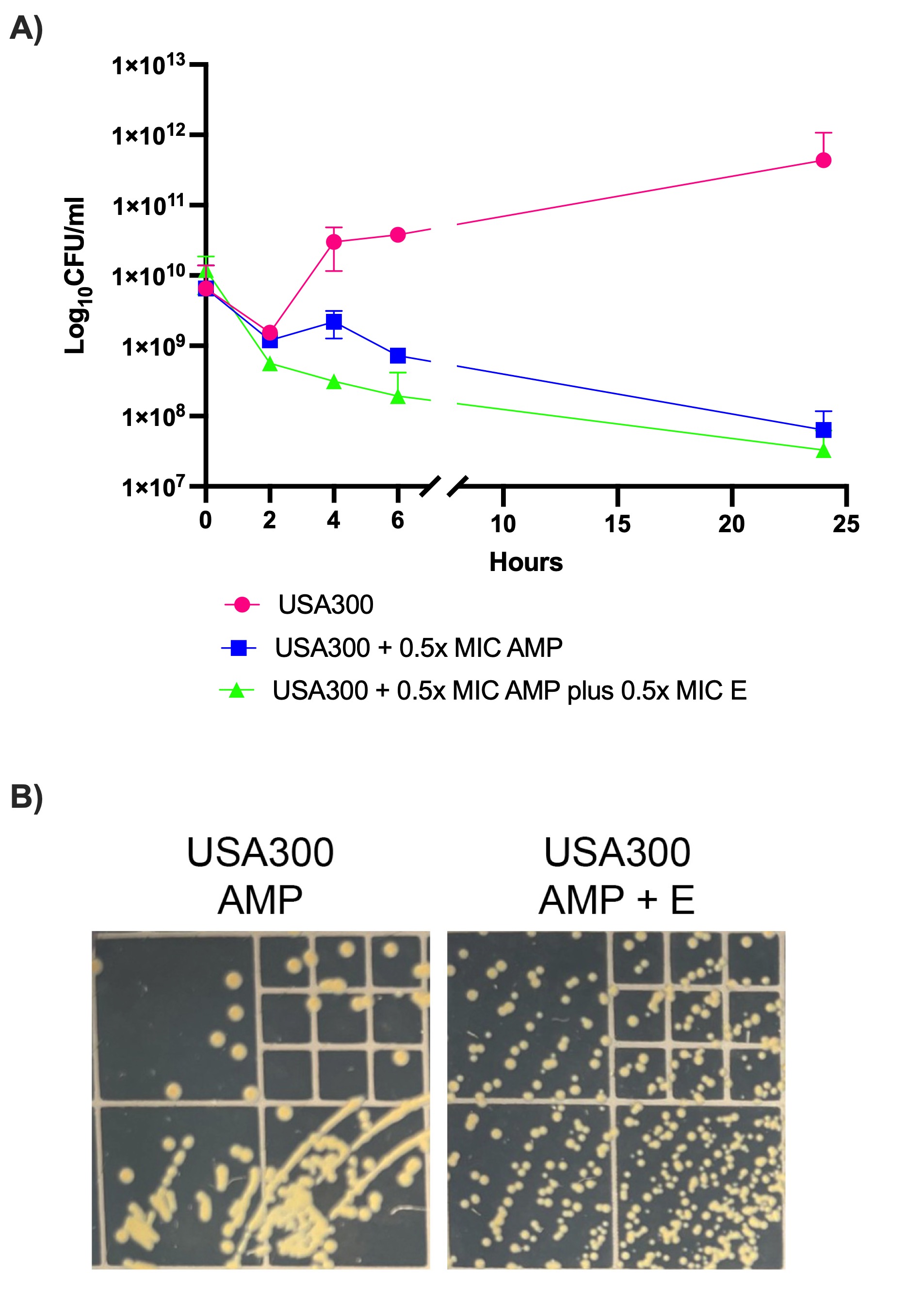

Supplement: Supplementary file 1 [file antibiotics-14-00812-s001.zip › Figure S2.jpg]
